# Pandemic Parliaments: Canadian Legislatures in a Time of Crisis

**DOI:** 10.1017/S0008423920000499

**Published:** 2020-05-15

**Authors:** Erica Rayment, Jason VandenBeukel

**Affiliations:** Department of Political Science, University of Toronto, Sidney Smith Hall, 100 St. George Street, Toronto, Ontario, M5S 3G3

## Abstract

Canadian legislatures’ responses to the COVID-19 pandemic have raised questions about whether and how parliaments should continue to meet during the pandemic (Reid, 2020; Thomas, 2020a). The purpose of this research note is twofold: (1) to document how Canadian legislatures have changed in response to the COVID-19 pandemic and (2) to assess the effect of these responses on legislatures’ ability to fulfill their core functions. Through an analysis of parliamentary records from all elected federal, provincial and territorial legislatures in Canada, we find that the role of parliaments as sites of citizen representation has suffered the most, whereas the scrutinizing and legislative functions of parliaments have tended to be preserved, albeit in a significantly truncated form. We argue that patterns in legislatures' varied responses to the pandemic reveal which aspects of parliamentary functioning these bodies de facto prioritize and which are at risk of being eroded.

## Introduction

Canadian legislatures’ responses to the COVID-19 pandemic have raised questions about whether and how parliaments should continue to meet during the pandemic (Reid, 2020; Thomas, 2020a). The purpose of this research note is twofold: (1) to document how Canadian legislatures have changed in response to the COVID-19 pandemic and (2) to assess the effect of these responses on legislatures’ ability to fulfill their core functions. Through an analysis of parliamentary records from all elected federal, provincial and territorial legislatures in Canada, we find that the role of parliaments as sites of citizen representation has suffered the most, whereas the scrutinizing and legislative functions of parliaments have tended to be preserved, albeit in a significantly truncated form. We argue that patterns in legislatures' varied responses to the pandemic reveal which aspects of parliamentary functioning these bodies de facto prioritize and which are at risk of being eroded.

## Conceptual Framework

Following Docherty (2005), we conceive of Canada's parliamentary institutions as designed to fulfill three core functions: representation, scrutiny, and legislation. Representation occurs in a variety of ways, but at its core entails “the making present *in some sense* of something which is nevertheless *not* present literally or in fact” (emphasis in original; Pitkin, 1967: 8–9). Legislatures fulfill their representative function insofar as they serve as venues in which citizens’ collective interests, local concerns, or party perspectives are made present in parliamentary proceedings by elected officials. Legislatures are also responsible for holding governments accountable for their actions and decisions. This scrutiny function is fulfilled by opposition members and government backbenchers in question period, through legislative debate, in caucus meetings, and through committees (Docherty, 2005: 16–18). Although “Westminster parliaments do not initiate legislation,” they play an important authorizing role in the legislative process, passing or defeating bills introduced by cabinet (Docherty, 2005: 19). This legislative function is fulfilled when members make the government's legislative intentions public through debate on the legislation, and occasionally modify legislation based on the input and perspectives raised in debate and committee.

## Data and Methods

To assess the extent to which these three functions of parliament have been affected, we gathered data for several key indicators: the number of meeting days since the declaration of an emergency, whether the meeting was conducted with a reduced number of legislators, whether the legislation passed, and whether question period has continued. We collected these data from a combination of each legislature's online records and available media reports, covering the period from the declaration of an emergency in each jurisdiction until April 30, 2020. We exclude the Senate because its functions as an unelected upper chamber differ from its elected counterpart (Smith, 2003).

The number of meeting days since the declaration of an emergency offers insight into all three functions. More frequent legislative meetings provide more opportunity for members to represent citizen concerns, scrutinize government actions, and provide input into the development of legislation. A reduced number of legislators in parliamentary meetings reduces the number and diversity of voices that can make citizens’ interests and concerns present in legislative proceedings and threatens the ability of legislatures to represent citizens. Question period is one of the most public ways in which legislatures hold executives to account (Docherty, 2005: 16). Its continuation, therefore, provides a clear indication of a legislature's ability to scrutinize the actions of the executive. The process by which legislation is passed also relates to a legislature's capacity for scrutiny. The passage of legislation with restricted time for debate severely undermines a legislature's ability to hold a government to account. Finally, the content of legislation itself provides an indicator of how important the legislative function is for a pandemic parliament. We review whether executives are seeking legislative authorization of emergency measures or if they are instead trying to bypass legislatures in order to respond quickly to the crisis.

## Results

Our results are summarized in Table 1. Following the declaration of an emergency, the number of sitting days in most legislatures has been limited. Of the 10 legislatures that met since the outbreak of the pandemic, six met only once. Alberta is an outlier, having met nine times since the declaration of a public health emergency on March 17. Alberta premier Jason Kenney argued the legislature should continue to sit as an essential service, and the provincial chief medical officer granted the legislature an exemption from a provincial ban on gatherings of more than 50 people (French, 2020). The federal House of Commons is the only legislature to have met virtually. It held three in-person meetings between March 24 and April 20, but beginning April 28 shifted to semiweekly online question periods in addition to weekly in-person sittings for core legislative functions (Aielo, 2020).

**Table 1:** Canadian legislatures and the COVID-19 pandemic to April 30, 2020

Legislature	Emergency declared	Meeting days since emergency	Reduced number of legislators	Question period	Legislative activity after emergency declaration
**Canada** **(House of** **Commons)**	Ontario emergency declared March 17	5	Yes	In person and virtual**	Parliament meets regularly to authorize emergency powers and spending measures through an expedited process, as well as a semiweekly virtual question period
**Ontario**	March 17	3	Yes	No	Passed a mini budget and COVID-19 related measures through an expedited process
**Quebec**	March 13*	1	Yes	No	Approved estimates and passed four bills initiated earlier in the session through an expedited process
**Nova Scotia**	March 22	0	*N/A*	*N/A*	*N/A*
**New Brunswick**	March 19	1	Yes	No	Passed COVID-19 related amendments to employment and emergency measures legislation through an expedited process
**Manitoba**	March 20	1	Yes	In person	Authorized emergency executive powers and spending measures through an expedited process
**British Columbia**	March 17*	1	Yes	In person	Passed worker protections and financial aid package through an expedited process
**Prince Edward Island**	March 16*	0	*N/A*	*N/A*	*N/A*
**Alberta**	March 17*	9	Yes	In person	Authorized financial aid, emergency powers, and ordinary legislation through a regular process
**Saskatchewan**	March 18	1	Yes	No	Met briefly to adjourn; emergency funds have been raised by special warrant, bypassing legislature
**Newfoundland and Labrador**	March 18*	1	Yes	No	Passed interim supply measures and COVID-19 related amendments through expedited process
**Northwest Territories**	March 19*	0	*N/A*	*N/A*	*N/A*
**Yukon**	March 18*	2	No	In person	Passed the budget (without COVID-19 related measures) through an expedited process
**Nunavut**	March 19*	0	*N/A*	*N/A*	*N/A*

* Indicates a public health emergency declaration rather than a provincial state of emergency.

** Technically, the House of Commons meetings after April 20 are not formal sittings but rather meetings of a COVID-19 special committee that includes all 338 MPs as well as a scheduled question period.

In every legislature except Yukon, meetings were held with a significantly reduced number of legislators to ensure compliance with physical distancing requirements. The scale of the reduction in the number of participating legislators is not available in all cases, but media reports and parliamentary records of participating speakers show that the scope of the reduction in participation has varied. In the House of Commons and the Quebec National Assembly, roughly 10 per cent of legislators attended emergency sessions, while in Alberta the government and opposition simply agreed “to have fewer than 50 MLAs and staff in the chamber at any given time” (Bellefontaine, 2020). Yukon did not reduce the number of participating legislators, but its only meetings took place on the two days immediately after a public health emergency was declared on March 18.

The focus of legislative activity undertaken in these modified parliamentary meetings fell into two main categories: (1) the passage of regular fiscal measures and (2) the passage of COVID-related emergency legislative changes. Fiscal measures included budgets and fiscal updates, approval of estimates, and the authorization of fiscal appropriations. Legislation in this category was passed in Alberta, Saskatchewan, Manitoba, Ontario, Quebec and Newfoundland. COVID-19 related emergency legislation included changes to employment law and emergency measures legislation in response to the impacts of the pandemic. Legislation in this category was passed federally and in British Columbia, Alberta, Manitoba, Ontario, New Brunswick, and Newfoundland. Of the 10 legislatures that met after the declaration of an emergency in their jurisdiction, only three (Yukon, Alberta, and Quebec) continued to pass regular legislation that did not fall under these two categories.

In most cases, the process through which legislation was passed was significantly expedited, with three readings and royal assent of bills all completed within a single day. In New Brunswick, legislation was introduced, passed, and given royal assent in an abbreviated legislative session that lasted less than 30 minutes. Media reports suggest the legislative changes were agreed to by the premier, key cabinet ministers, and leaders of the opposition parties prior to the legislative sitting (Poitras, 2020), but the legislature's scrutiny role was nonetheless bypassed. In addition to a truncated process for passing legislation, half of the legislatures that met after the declaration of an emergency jettisoned the usual elements of legislative proceedings, including question period. Only Yukon, British Columbia, Alberta, Manitoba and the House of Commons have retained the opportunity for legislators to hold the government to account through question period.

Some committee meetings have continued in British Columbia, Quebec, and the federal parliament as an alternate site of representation and scrutiny. However, most committee meetings have been virtual rather than in person, and for the most part directed specifically at scrutinizing government responses to the COVID-19 pandemic.

## Discussion

These results imply several key conclusions about the effects of adaptations in response to the COVID-19 pandemic on legislatures’ ability to fulfill their core functions and which functions these institutions de facto prioritize. First, we can see that legislatures’ representation function has suffered the most during the pandemic. Nine of the 10 legislatures that met after an emergency was declared substantially reduced the number of legislators in attendance. Party leaders have been empowered to select which legislators are able to attend, undermining the ability of individual legislators to participate and affect outcomes. Furthermore, data taken from the federal parliament suggests this has led to a severe regional imbalance (Thomas, 2020b). Similarly, the reduction in sitting days, which reduces the risk of pandemic spread by allowing legislators to stay home, also limits opportunities for representation.

Legislatures’ ability to scrutinize executive action has also suffered to a lesser extent. The House of Commons is providing regular opportunities for MPs to scrutinize the government's response through a reduced sitting every Wednesday and a twice-weekly virtual question period (Aielo, 2020). Scrutiny of the federal government has had meaningful outcomes, notably when the government's attempts to authorize the finance minister to raise taxes without parliamentary approval were significantly limited by the opposition (Tasker, 2020). The record in the provinces is mixed: brief opportunities for questions were provided in Manitoba, British Columbia, and Yukon. Alberta MLAs had regular opportunities for questions while the legislature continued to meet until the middle of April. In the rest of the provinces, opportunities for legislative scrutiny were bypassed in favour of expeditiously passing emergency legislation.

The function that has been best preserved since the declaration of an emergency is legislation, albeit with significant limitations. Governments scrambled to pass several bills throughout the middle of March, many of which were in direct response to the pandemic. Since then, numerous legislatures have reconvened to pass various emergency response measures through expedited processes. The reason for this is simple: the executive needs legislative approval to raise money and amend existing legislation. The quality of these post-pandemic legislative processes is questionable: in most cases, major emergency response measures were passed in one sitting, with virtually no opportunity for discussion, input, or oversight. In some jurisdictions, the executive has sought to circumvent even this minimal legislative function. In Saskatchewan, the government is raising and spending billions of dollars through special warrants, rather than with legislative approval (Bamford, 2020). Similarly in Yukon, the legislature has been bypassed in favour of orders-in-council (Windeyer, 2020).

Canada's legislatures have responded to the COVID-19 pandemic in a way that has left little room for their representative function. This is perhaps understandable, given the need for governments to respond quickly to the crisis. However, we have also seen that the ability of legislatures to scrutinize and authorize the actions of the executive through legislation has been substantially undermined. As the crisis looks likely to continue for the foreseeable future, Canadians should consider the ways in which we can reclaim a constructive role for legislatures in a time of crisis.
